# Supplementary material for: Successional Shifts in Soil C:N:P stoichiometry and its environmental controls during forest restoration in Huize County, Qujing, Yunnan Province
Source: PLoS One. 2026 Aug 21;21(8):e0356261. doi: 10.1371/journal.pone.0356261 (PMC13496410; doi:10.1371/journal.pone.0356261)
Supplement: Supplementary file 2 [file pone.0356261.s004.zip › Data and Code/RDA/evergreen broadleaf forest/RDA-2.pdf]

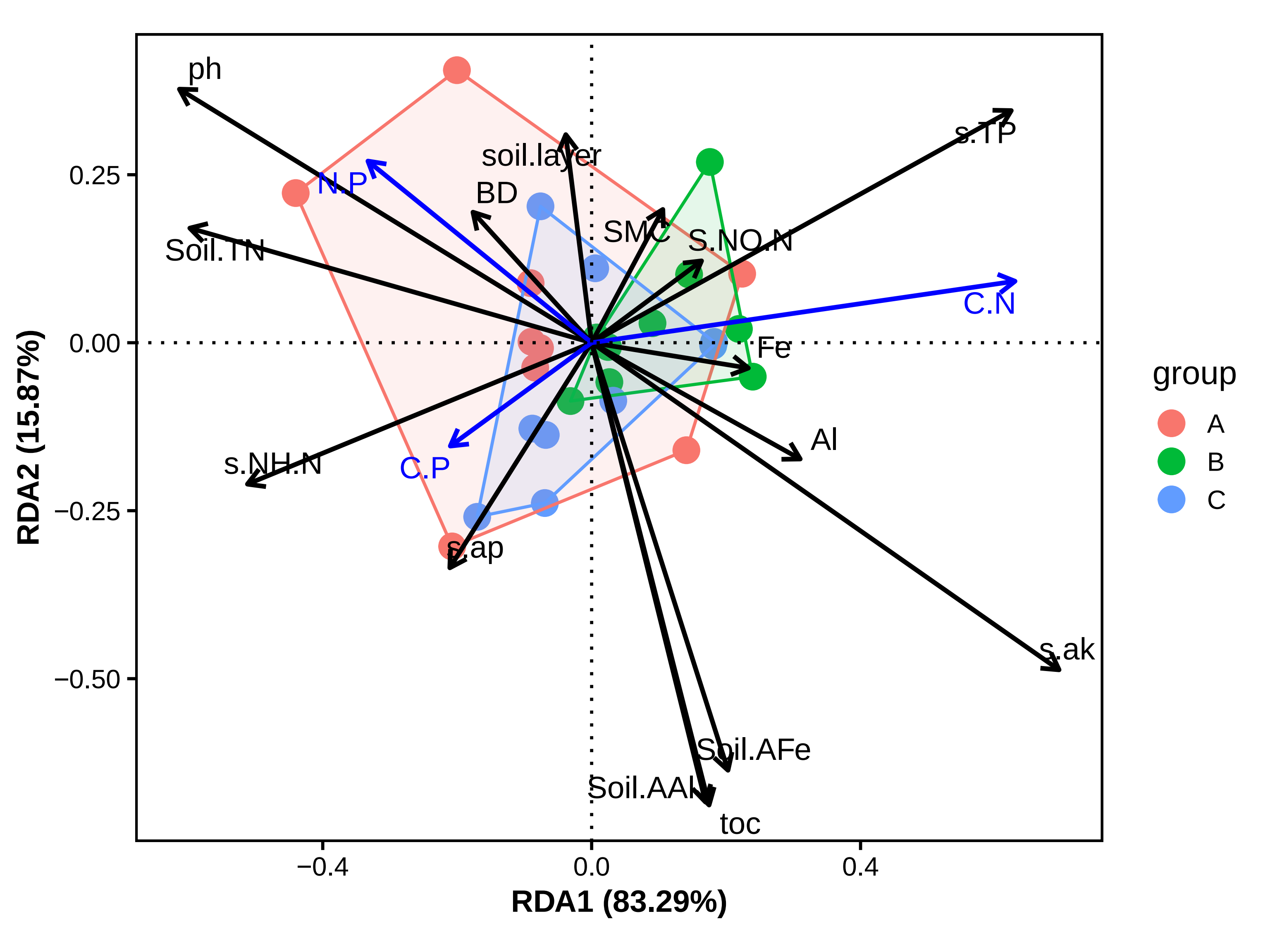

ph
s.TP
soil.layer
N.P
BD
SMC
S.NO.N
Soil.TN
C.N
Fe
Al
s.NH.N
C.P
s.ap
s.ak
Soil.AFe
Soil.AAl
toc
0.25
0.00
−0.25
−0.50
−0.4
0.0
0.4
RDA1 (83.29%)
RDA2 (15.87%)
group
A
B
C
